# Oregano Oil, Epsilon-Polylysine and Citric Acid Assisted Inactivation of *Salmonella* in Two Kinds of Tahini during Thermal Treatment and Storage

**DOI:** 10.3390/foods10061272

**Published:** 2021-06-03

**Authors:** Yuanmei Xu, Xiangyu Guan, Biying Lin, Rui Li, Shaojin Wang

**Affiliations:** 1 College of Mechanical and Electronic Engineering, Northwest A&F University, Yangling 712100, China; yuanmeixu@nwafu.edu.cn (Y.X.); xiangyuguan@nwafu.edu.cn (X.G.); linbiying@nwafu.edu.cn (B.L.); ruili1216@nwafu.edu.cn (R.L.); 2 Department of Biological Systems Engineering, Washington State University, Pullman, WA 99164-6120, USA

**Keywords:** *Salmonella montevideo* CICC21588, tahini, natural antimicrobials, thermal inactivation, antimicrobial effects

## Abstract

Tahini and tahini-based products are popular with consumers due to their special flavor and high nutritional values, but often have been linked to *Salmonella* outbreaks. The objective of this study was to compare effects of different kinds of natural antimicrobials on *Salmonella* inactivation in undiluted and diluted tahini during thermal treatment and storage. Results showed that the Weibull model was more suitable to describe the thermal inactivation behavior of *S. montevideo* CICC21588 in two kinds of tahini than the first-order model. Inactivation curves were concave-upward in undiluted tahini but concave-downward in diluted tahini. During storage of undiluted tahini, 3% oregano oil caused extra 1.44 or 0.80 log CFU/g reductions after 7 days at 25 °C or 4 °C compared to the control and 0.5% citric acid caused an extra reduction of 0.75 log CFU/g after 7 d at 4 °C. For diluted tahini, 2–3% oregano oil and 0.4–0.5% ε-polylysine reduced more populations compared to undiluted tahini. These antimicrobials all inhibited the growth of *S. montevideo* during 24 h at 25 °C and ε-polylysine had the best effect. Furthermore, these antimicrobials enhanced the *Salmonella* inactivation in diluted tahini during thermal treatment, and there was less of a synergistic effect of thermal and antimicrobials in undiluted tahini due to less sublethal injured cells caused by heat. This study may provide useful information for *Salmonella* inactivation in tahini.

## 1. Introduction

Tahini (sesame seeds paste) is a well-known ready-to-eat product in Middle Eastern and Eastern Mediterranean countries due to its special flavor and high nutritional value [1]. It has 57–65% fat, 6.4–9.0% carbohydrate, 23–27% protein and low water content (<1%) [2]. Tahini not only can be consumed directly, but also mixed with other ingredients to make tahini-based products, such as hummus, halva, baba ghanoush and various salad dressings [3,4,5]. In Chinese diet, tahini has been usually diluted with 1–3 times water or adding other components according to personal tastes to make noodle dip or hot pot dip [6].

**Figure 1:**
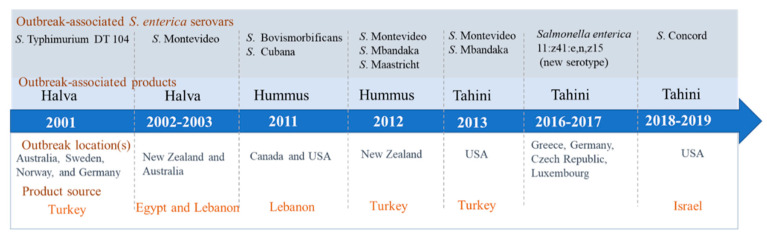
*Salmonella* outbreaks associated with the tahini or tahini-based products.

However, a number of *Salmonella* illness outbreaks and recalls related to the tahini and tahini-based products consumption happened at 2001 [7], 2002–2003 [8], 2011 [9], 2012 [10], 2013 [11], 2016–2017 [12], and 2018–2019 [13,14], resulting in a serious economic loss and posing a big threat to people’s lives. Figure 1 shows the *Salmonella* illness outbreaks happened at 2001–2019, including *Salmonella* serotypes, outbreak locations, associated products, and product sources.

Generally, the production of tahini involves soaking, dehulling, roasting, and milling of sesame seeds [15] and the roasting treatment (110~150 °C for 30–60 min) is sufficient to inactivate most food pathogens [16]. However, some bacteria might survive at the roasting process due to the insufficient heating and the increase of thermal resistance of bacteria on sesame seeds with low water activity (a_w_) [16,17]. In addition, contamination might occur during the milling or packaging process after thermal treatment. Although *Salmonella* cannot grow in tahini due to its low a_w_ (0.16–0.25), the high fat content may help them persist for a long period in low a_w_ environment [15,17] and also improve their survival rate in gastrointestinal environment, resulting in a reduction of the dose–response curve [18]. Furthermore, tahini is used commercially as a common ingredient to prepare ready-to-eat tahini-based products or at home or restaurant to prepare diluted tahini by adding different proportions of water as needed. These products are not consistently refrigerated and may be held at room temperature for several hours. High water content and nutrition in these tahini-based products provide a suitable environment for pathogens to grow, posing a threat to consumer health. Therefore, it is necessary to explore effective methods to control *Salmonella* contamination in tahini or tahini-based products.

In recent years, the interest in using natural antimicrobials to control pathogens in the food industry has increased due to their safety. Essential oils (EOs), ε-polylysine (ε-PL) and organic acid, have been widely studied in many food products to control the growth of pathogens, such as fresh lettuce [19], beef [20], milk [21], almond and pine nut kernels [22]. Currently, the most undiluted tahini currently in the market contains no antimicrobials, but tahini-based products often contain artificial preservatives. Therefore, it is necessary to investigate the potential applications of natural antimicrobials to control pathogens in tahini or tahini-based products during storage. Furthermore, natural antimicrobials have been widely applied to assist microbial inactivation during thermal processing of various products to save heating time or guarantee food quality [23]. However, limited information is available on the pathogen inactivation of natural antimicrobials during thermal treatments of tahini and tahini-based products.

The objectives of this study were (1) to determine thermal inactivation kinetics of *Salmonella* in undiluted or diluted tahini, (2) to evaluate the sublethal injured cells by thermal treatments in undiluted or diluted tahini, (3) to evaluate the inhibitory effects of oregano oil, ε-PL and citric acid in undiluted or diluted tahini during storage and (4) to evaluate the synergistic effects of heating and antimicrobials in undiluted or diluted tahini.

## 2. Materials and Methods

### 2.1. Salmonella Strains and Culture Preparation

Four *Salmonella* enterica serotypes were used in this study. *Salmonella montevideo* CICC21588 was obtained from the China Center of Industrial Culture Collection. *Salmonella typhimurium* CMCC50115 was obtained from National Center for Medical Culture Collections. *Salmonella mbandaka* NYS5-8 and *Salmonella enteritidi* R8-1-1 were provided by the College of Food Science and Engineering, Northwest A & F University, Yangling, China. All strains were stored at −20 °C with 20% glycerol. Prior to the experiment, one loopful of frozen culture was streaked onto tryptic soy agar (TSA; Beijing Land Bridge, Beijing, China) plates and incubated at 37 °C for 24 h. A single colony was transferred to 30 mL of tryptic soy broth (TSB; Beijing Land Bridge, Beijing, China) and incubated at 37 °C for 24 h. The 3 mL of culture was transferred to 300 mL of fresh TSB and incubated at 37 °C to reach the stationary phase. The strain cultures were centrifugated (4000× *g* for 10 min) and the cell pellets were resuspended using 20 mL of phosphate buffer saline (PBS, 0.01 M, pH 7.2), then centrifugated again and finally resuspended with 300 μL of PBS to obtain a required concentration of 10^10^–10^11^ CFU/mL.

### 2.2. Sample Preparation

Tahini (fat 52.6%, protein 26% and carbohydrate 15.7%) was used in this study and the indigenous microbiota in tahini was less than 10^2^ CFU/g, which did not interfere with the count of *Salmonella* cells grown on TSA. Diluted tahini (50% *w*/*w*) was prepared by adding sterilized water, simulating common edible methods at home or restaurant in the Chinese diet [6].

Essential oils (EOs, Jiangxi Global Natural Spice Co., Ltd., Jiangxi, China) including oregano, cinnamon, thyme, tea tree, eucalyptus, peppermint, garlic and turmeric oils were directly added into undiluted and diluted tahini, obtaining the concentration of 1–3%. ε-PL (BioDuly, Nanjing, China), tea-polyphenol (Macklin, Shanghai, China) and citric acid (CA, Huada, Guangdong, China) were dissolved or diluted to reach appropriate concentrations using sterilized water before adding to samples, obtaining the final concentration of 0.05–0.5% for organic acid and 0.05–0.4% for ε-PL or tea-polyphenol. The pH values of samples were measured at room temperature using a pH meter (PHS-25; INESA, Shanghai, China). 

### 2.3. Thermal Treatment of S. montevideo CICC21588 in Undiluted or Diluted Tahini

The thermal treatment was conducted using a heating block system (HBS) as described in a previous study [24]. About 0.8 g undiluted or diluted tahini was put into the sterile thermal death test (TDT) cells and 8 μL of *Salmonella* suspension was inoculated on samples to achieve a cell density of about 10^8^–10^9^ CFU/mL. To study the *Salmonella* inactivation of EOs, ε-PL and organic acid during heating treatments, antimicrobials were added to the cells to obtain the target concentrations (oregano of 1–3%, citric acid of 0.05–0.5% and ε-PL of 0.05–0.4%). The a_w_ of control group samples was adjusted by adding the same amount of deionized water as treated samples, but without antimicrobials.

Five TDT cells filled with 0.8 g inoculated samples were heated to the target temperatures (75 °C, 85 °C and 95 °C for undiluted tahini; 56 °C, 58 °C and 60 °C for diluted tahini) with a heating rate of 5 °C/min using HBS, which was used to simulate the fast-heating rate in radio-frequency treated products. When the sample temperatures reached the target set-point, one cell was removed and defined as a time-zero sample. All the test cells were immediately cooled using ice-water after heat treatment.

### 2.4. Oxford Cup Assay of Antimicrobials

To study antibacterial effects of these natural antimicrobials against *Salmonella* of different serotypes rather than one specific serotype, *Salmonella montevideo* CICC21588, *Salmonella typhimurium* CMCC50115, *Salmonella mbandaka* NYS5-8 and *Salmonella enteritidi* R8-1-1 were used and compared. Antimicrobial activity of EOs, ε-PL, tea polyphenol and organic acid against *Salmonella* was determined by oxford cup assay (ID of 6 mm and OD of 8 mm) according to Bian et al. [25] with some modifications. Briefly, oxford cups were put on the agar plates (2% *w*/*v* agar) and then TSB with 0.75% agar containing *Salmonella* (10^6^ CFU/mL) was poured onto the plates and allowed to solidify. Afterwards, 100 μL EOs or 200 μL of two concentrations (5% and 10%) of ε-PL, tea polyphenol and organic acid were added into the oxford cups. The plates were incubated at 37 °C for 24 h and the inhibitory zone diameters were determined.

### 2.5. Control of S. montevideo CICC21588 in Undiluted or Diluted Tahini by Antimicrobials 

Oregano oil was added to undiluted tahini to obtain the final concentrations of 1% and 3% (*v*/*w*). For water soluble antimicrobials, 20% (*w*/*v*, clear solution) or 40% (*w*/*v*, suspension) of ε-PL and 80% CA (*w*/*v*) were added to undiluted tahini for obtaining final concentrations of 0.2% or 0.4%, and 0.3% or 0.5% (*w*/*w*), respectively. The a_w_ of control group samples was adjusted to the same with that of the treated samples. Afterwards, samples were inoculated with *Salmonella* suspension, obtaining a cell concentration of 10^8^–10^9^ CFU/g and stored at 25 °C or 4 °C for 7 days. *S. montevideo* CICC21588 populations in inoculated samples were enumerated after 0, 1, 2, 3 and 7 days. 

Oregano oil was added to diluted tahini to obtain final concentrations of 1–3% (*v*/*w*). For water soluble antimicrobials, 20% ε-PL (*w*/*v*) and 20% CA (*w*/*v*) were added to diluted tahini to obtain final concentrations of 0.05–0.5% (*w*/*w*). Then samples were inoculated with *Salmonella* suspension, obtaining a cell concentration of 10^6^ CFU/g and stored at 25 °C or 4 °C for 7 days. Bacterial enumeration was conducted at 0 h, 4 h, 7 h, 11 h, 14 h, 24 h, 36 h, 52 h and 72 h and after 7 days.

### 2.6. Bacterial Enumeration

After the treatment, each sample was transferred into a sterile tube, diluted using 9 times sterile PBS and shaken for 1 min. The suspension was serially diluted 10-fold in sterile PBS and 0.1 mL of diluent was spread onto TSA, and incubated at 37 °C for 24–48 h until the colonies were formed. To evaluate the sublethal injured cells, xylose lysine deoxycholate (XLD, Hopebio, Qingdao, China) agar plate [26] and TSA plate containing 4% NaCl (TSA-NaCl) [27] were used. XLD containing 0.25% deoxycholate and TSA with 4% NaCl (TSA-NaCl) plates have been used to evaluate outer membrane damage and cytoplasmic membrane damage of *Salmonella*, respectively [28], because cells with membrane damages cannot grow on XLD and TSA-NaCl plates but grow on TSA. However, this method could only roughly reflect sublethal cell level but was not completely accurate, because some cells with small damage could still grow on the selective medium.

### 2.7. Modeling of Thermal Inactivation Kinetics

The first-order kinetic and Weibull distribution models were used to fit the thermal inactivation data. The first-order kinetic model was defined by the following equation:

(1) $$\log\;S\left(t\right)\frac{N}{N_{0}}=-\frac{t}{D}$$

where *S(t)* is the survival ratio, *N* (CFU/g) represents the bacteria population at the time *t* (min) of isothermal treatment, *N*_0_ (CFU/g) represents the bacteria population at the time reaching the target temperature and D is the decimal reduction time (min) at temperature T (°C). 

The Weibull model can be expressed as:

(2) $$\log\;S\left(t\right)=-{\left(\frac{t}{\delta }\right)}^{p}$$

where *δ* is scale parameter and *p* is shape parameter, indicating the survival curve’s concavity. *p* = 1, <1 and >1 represent liner, concave-upward (tailing) and concave-downward (shoulder) inactivation curves, respectively. For both the first-order kinetic and the Weibull distribution models, the coefficient of determination (R^2^) reflects the goodness of fit.

### 2.8. Statistical Analysis

All treatments were conducted in triplicate. The statistical significance of differences was evaluated by a one-way analysis of variance (ANOVA) and the Duncan post-hoc test (*p* = 0.05) using the SPSS statistics 17.0 software (SPSS Inc., Chicago, IL, USA). The parameters for the first-order kinetic and Weibull distribution models were obtained using the SPSS Statistics 17.0. 

## 3. Results and Discussion

### 3.1. Thermal Inactivation of S. montevideo CICC21588 in Undiluted or Diluted Tahini

**Figure 2:**
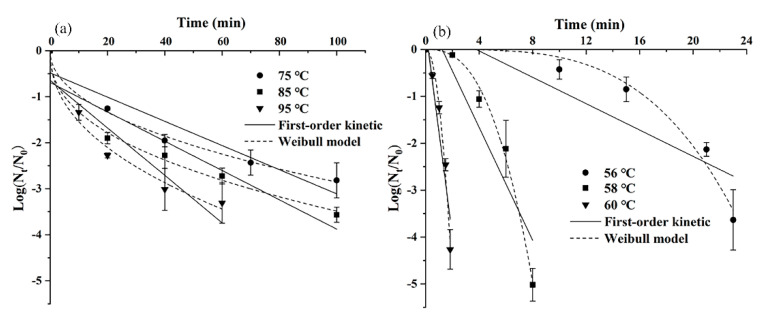
Thermal inactivation kinetics of *S. montevideo* CICC21588 in undiluted (a_w_ = 0.256) (**a**) and diluted tahini (a_w_ = 1.000) (**b**) at different temperatures.

*S. montevideo* CICC21588 was selected to be further used in tahini samples due to its most desiccation and thermal resistance in undiluted tahini among four strains of serotypes according to results of pre-experiments (data not shown). The thermal inactivation curves of *S. montevideo* CICC21588 in undiluted or diluted tahini with a heating rate of 5 °C/min in the HBS under different temperatures are shown in Figure 2. D-, δ- and *p*-values obtained from first-order kinetic and Weibull distribution models for describing the thermal inactivation behavior are listed in Table 1. The larger coefficient of determination (*R*^2^ = 0.973–0.996) and lower root mean square deviation (RMSE = 0.078–0.279) of the Weibull model compared to those (*R*^2^ = 0.775–0.906, RMSE = 0.435–0.913) of the first-order kinetic model indicated that the Weibull model was more suitable to describe the thermal inactivation behavior of *S. montevideo* CICC21588 in undiluted and diluted tahini. In undiluted tahini, *δ*-values were 10.6, 5.21 and 3.84 at 75 °C, 85 °C and 95 °C, respectively. The high thermal resistance of *Salmonella* was reported in undiluted sample due to its extremely low a_w_ (0.256) and high fat content (52.6%). He et al. [29] also found minimum times to achieve 1-log reduction of *S. enterica* serotypes in peanut butter (a_w_ 0.2, fat 49%) at 90 °C were 6.32–12.08 min based on the Weibull model. Krapf and Gantenbein-Demarchi [30] studied the thermal inactivation of *Salmonella* in dark chocolate (a_w_ 0.3–0.5, fat ≥ 18%) and found that the D-value at 90 °C was 25 min. In diluted tahini, *δ*-values were 16.52, 4.38 and 0.92 at 56 °C, 58 °C and 60 °C, respectively. *δ*-value decreased with the increase of temperature and a_w_, similar to results obtained by Gautam, Govindan, Gänzle and Roopesh [31].

In undiluted tahini (a_w_ = 0.256), *p*-values of the Weibull model were less than one under three thermal treatments of 75 °C, 85 °C and 95 °C. The inactivation curves were concave-upward, as shown in Figure 2a, in accordance with previous reports, such as thermal inactivation of *Salmonella* in peanut butter [32], black peppercorns, pecans, almonds [33] and pet food pellets [31]. According to these reports, thermal inactivation curves of pathogens in low moisture foods (LMFs) usually exhibit concave-upward, indicating that pathogens are inactivated directly without a large damage accumulation and the survival populations become increasingly resistant to thermal stress.

As shown in Figure 2b, a shoulder was observed for the inactivation curves of *S. montevideo* CICC21588 in diluted tahini by heating at 56 °C, 58 °C and 60 °C. The concave-downward thermal inactivation curves also appear in foods with high water content, such as *Salmonella* inactivation in whole liquid egg [34], *Escherichia coli* O157:H7 inactivation in orange juice [35] or apple juice [28] and *Listeria monocytogenes* inactivation in semi-skim milk [36]. These results indicated that bacteria cells were firstly resistant to the sublethal temperatures but continuous heating made the injured cells difficult to survive.

### 3.2. The Sublethal Injured Cells in Thermal Treatments in Undiluted and Diluted Tahini

**Figure 3:**
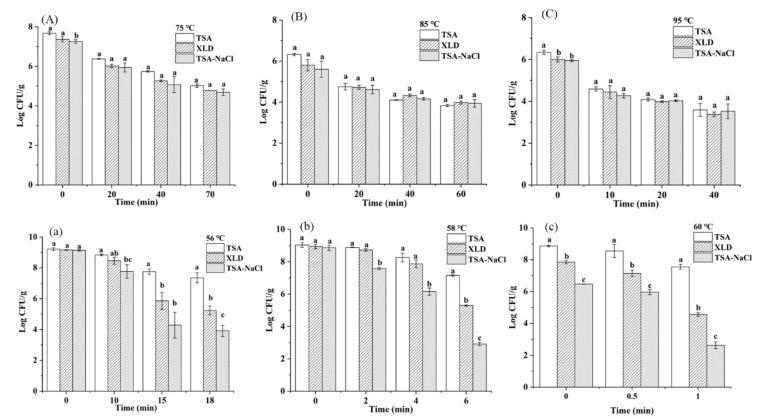
Population of *S. montevideo* CICC21588 in undiluted tahini (**A**–**C**) and diluted tahini (**a**–**c**) grown on TSA, XLD and TSA-NaCl plates at different treatment temperatures. The same low case letter among different plates at each treatment indicates values are not significantly different (*p* > 0.05).

Figure 3 presents the levels of sublethal injured *S. montevideo* CICC21588 cells in undiluted or diluted tahini by thermal treatments. For undiluted tahini, as shown in Figure 3A–C, statistically significant differences of the population grown on the TSA, XLD and TSA-NaCl were only observed at the thermal treatments of 75 °C for 0 min and 95 °C for 0 min (*p* < 0.05), indicating that cell membrane damages occurred under these treatments. However, the injured cell populations were less than 1 log CFU/g. The similar result was also obtained by Lee et al. [37], who found that steam treatment (93 °C) inactivated *Salmonella enteritidis* on the raw shelled almonds without causing significant sublethal injury.

For diluted tahini, a large number of injured cells were produced under the thermal treatments of 56 °C, 58 °C and 60 °C, as shown in Figure 3a–c. The heat treatments of 56 °C for 18 min, 58 °C for 6 min and 60 °C for 1 min caused more than 3 log CFU/g injured cells. In addition, the lower population grown on the TSA-NaCl than on the XLD after three temperature treatments for each time indicated that the thermal stress caused more damage to cytoplasmic membrane than outer membrane of *S. montevideo* CICC21588. It has been reported that a mild thermal stress (54–60 °C) causes a large number of injured cells of *Escherichia coli* O157:H7 in fruit juice, however, more outer membrane damages are observed than cytoplasmic membrane damages, which is inconsistent with the results in this study possibly due to the different strain used [28,35].

That more sublethal injured cells caused by thermal stress in diluted tahini than in undiluted tahini corresponds to the thermal inactivation curves as described in Section 3.1, *S. montevideo* CICC21588 populations were inactivated directly by relatively high temperatures in undiluted tahini without causing significant injured cells and curves were concave-upward. While in diluted tahini, a damage accumulation was observed at the beginning of heating and the inactivation curves were concave-downward.

### 3.3. Inhibitory Activity against Salmonella of EOs, ε-PL, Tea Polyphenol or Organic Acid

Table 2 and Table 3 show the inhibitory activity against *Salmonella* of EOs, ε-PL, tea polyphenol and organic acid using the oxford cup assay. For EOs, as listed in Table 2, tea tree, eucalyptus, peppermint, garlic and turmeric oils exhibited less inhibitory activity or even no effect against four *Salmonella* serotypes compared to oregano, cinnamon and thyme oils. Furthermore, oregano oil exhibited the strongest antimicrobial activity with inhibition zones of 21.38–24.00 mm. 

For water-soluble antimicrobial agents, as presented in Table 3, ε-PL and tea polyphenol also had the inhibitory activity against *Salmonella* with inhibition zones of 16.25–23.50 mm and 13.00–18.50 mm at the concentration of 10%, respectively. 

Organic acids, such as citric acid and lactic acid, are widely used in the food industry to control pathogens or regulate acid flavor, generally regarded as safe [38]. From Table 3, citric acid and lactic acid both exhibited significant inhibitory effect against *Salmonella* with inhibition zones of 25.75–27.70 mm and 24.00–27.20 mm at the concentration of 10%, respectively. The antimicrobials effect of lactic acid was slightly smaller than that of the citric acid. Therefore, oregano oil, ε-PL, tea polyphenol and CA were further selected to control *S. montevideo* CICC21588 in undiluted and diluted tahini according to the inhibition zones.

### 3.4. Effect of Oregano Oil, ε-PL and CA on the Viability of S. Montevideo CICC21588 in Undiluted and Diluted Tahini

**Figure 4:**
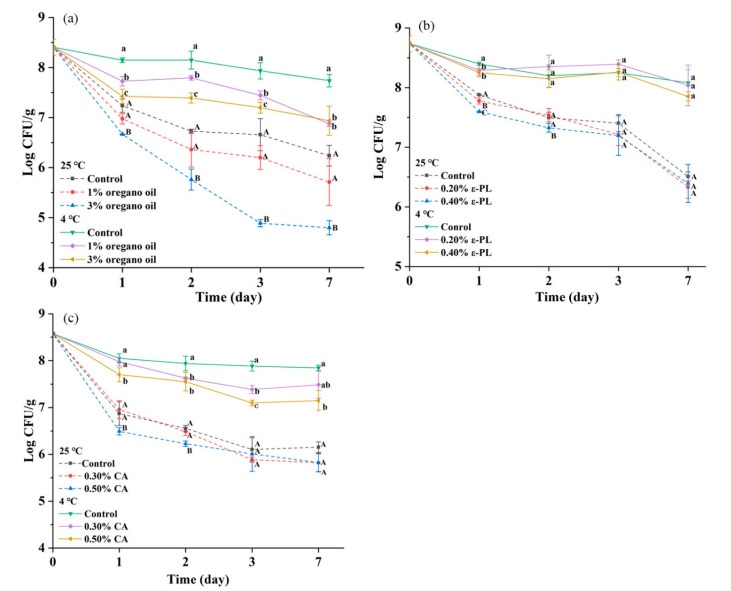
Antimicrobial activity of oregano oil (**a**), ε-PL (**b**) and CA (**c**) against *S. montevideo* CICC 21588 in undiluted tahini at 25 °C or 4 °C. The same lower case letter among different treated samples at each day during 4 °C storage indicates mean values are not significantly different (*p* > 0.05). The same uppercase letter among different treated samples at each day during 25 °C storage indicates values are not significantly different (*p* > 0.05).

As shown in Figure 4a, *S. montevideo* CICC21588 population was significantly reduced by 3.61 log CFU/g in undiluted tahini with 3% oregano oil after 7 days at 25 °C, compared to 2.17 log CFU/g in the control (*p* < 0.05). After 7 days at 4 °C, 1.53 and 1.47 log CFU/g reductions were observed in undiluted tahini with 1% and 3% oregano oil, respectively, compared to 0.67 log CFU/g in the control (*p* < 0.05). This result indicated that 3% oregano oil could effectively inactivate *Salmonella* in undiluted tahini without bringing water into samples, causing no influence on its shelf life. However, 3% oregano oil influenced the flavor of tahini and might not be accepted by consumers. 

In addition, results showed that the *Salmonella* population in undiluted tahini declined faster at higher temperatures during the storage period of 7 d, in accordance with previous research related to LMFs [39]. Park, Oh and Kang [40] found that the survival of *S*. Tennessee cells in peanut butter was significantly lower at 22 °C, compared to 4 °C during the storage period of 14 d. The possible reason probably is that the metabolism of bacteria at the relatively low temperature is lower than that in the high temperature, which helps *Salmonella* to maintain biological activity.

The addition of ε-PL and CA may bring water into undiluted tahini, which might accelerate the oxidative rancidity of fat, influencing its shelf life. Therefore, it is necessary to ensure the original concentrations of ε-PL and CA high. From Figure 4c, 0.2–0.4% ε-PL had no effect on the *Salmonella* reduction during the storage period of 7 days at 4 °C or 25 °C (*p* > 0.05). A reduction of 1.42 log CFU/g was observed in undiluted tahini with 0.5% CA after 7 days at 4 °C, compared to the control of 0.73 log CFU/g (*p* < 0.05). This result indicated that 0.5% CA only caused less than 1 log CFU/g extra reduction compared to the control after 7 days of storage period. Although Al-Nabulsi et al. [41] have found more pronounced bactericidal effect against *Salmonella typhimurium* in undiluted tahini by CA compared to our results, but the amount of water brought into tahini is not shown in their study, which would influence the bactericidal effect.

**Figure 5:**
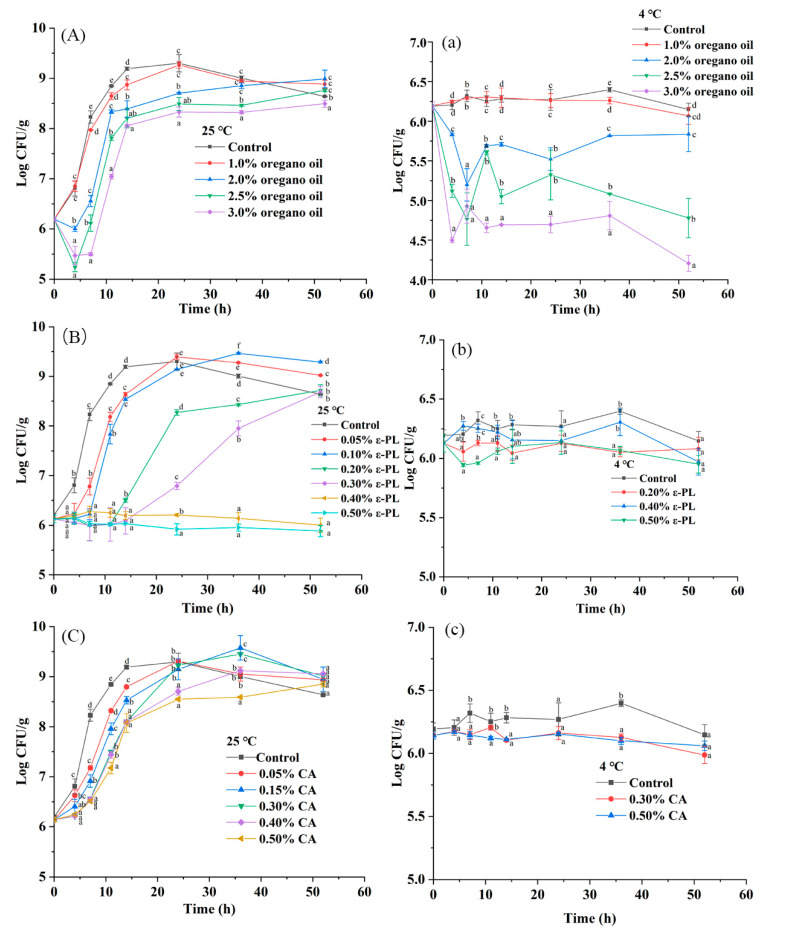
Antimicrobial activity of oregano oil, ε-PL and CA against *S*. *montevideo* CICC21588 in diluted tahini at 25 °C (**A**–**C**) or 4 °C (**a**–**c**). The same lower case letter among different treated samples at each day during storage indicates mean values that are not significantly different (*p* > 0.05).

The inhibitory effects of oregano oil, ε-PL and CA against *S. montevideo* CICC21588 in diluted tahini during the storage period of 7 days at 4 °C or 25 °C are shown in Figure 5 and Table 4. At 25 °C, the bacteria grown from 6.19 log CFU/g up to the maximum population of 9.30 log CFU/g at 24 h, then slightly decreased to 8.63 log CFU/g at 52 h and 8.70 log CFU/g at 72 h in diluted tahini without any antimicrobials, indicating that 50% (*w/w*) diluted tahini provided a suitable environment for *Salmonella* growth, posing a great threat to consumer health. After 7 days at 25 °C, samples without antimicrobials suffered deterioration caused by *Bacillus subtilis* originally existed in tahini (characterized by 16S rRNA gene sequencing), influencing the *Salmonella* enumeration. During the storage period of 7 days at 4 °C, *S. montevideo* CICC21588 population remained stable, about 6.19–6.40 log CFU/g in diluted tahini without antimicrobials.

As shown in Figure 5A and Table 4, the addition of 2–3% oregano oil to diluted tahini slowed down the growth of *S. montevideo* CICC21588 and slightly reduced the population at 4 h. 3% oregano oil could inhibit the growth up to 7 h. After 2 or 3 days at 25 °C, the population reached levels of 8.99 or 8.67 log CFU/g with 2% and 3% oregano oil, respectively. While after 7 days at 4 °C, 2–3% oregano oil significantly reduced *S. montevideo* CICC 21588 population by 0.70–3.57 log CFU/g (*p* < 0.05) as listed in Table 4. Furthermore, diluted tahini samples with 1–3% oregano oil did not suffer deterioration, suggesting that oregano oil could inhibit the growth of *Bacillus subtilis.*

Results also showed that oregano oil was more effective in diluted tahini than in undiluted tahini at 4 °C. In undiluted tahini, 1.47 Log CFU/g reduction was observed with 3% oregano oil compared to 0.67 log CFU/g reduction in the control, while 3.57 log CFU/g reduction in diluted tahini compared to no reduction in the control after 7 days at 4 °C. Diluted tahini used in this study had less fat, more water content and better fluidity than undiluted tahini. It is reported that increased fat content has a negative effect on the antimicrobial efficacy of EOs in model foods, such as peanut paste [42], soft cheese with different fat content [43] or model media containing different concentrations of sunflower oil [44]. In addition, water content is also a nonnegligible factor because water facilitates the movement of antimicrobial compounds to the target site in the bacterial cell [42]. 

As shown in Figure 5B and Table 4, the antimicrobial activity of ε-PL in diluted tahini depended on its concentration at 25 °C. ε-PL inhibited the growth of *S. montevideo* CICC21588 for 4 h with 0.05% ε-PL and for 72 h with 0.5% ε-PL. However, after 7 days at 25 °C, the populations reached the maximum numbers of approximately 9 log CFU/g. While at 4 °C, *S. montevideo* CICC21588 population remained stable for 72 h, about 6 log CFU/g with the addition of 0.05–0.5% ε-PL (Figure 5b) and after 7 days, the population was reduced to 4.59 and 3.68 log CFU/g with 0.4% and 0.5% ε-PL, respectively (Table 4). Furthermore, diluted tahini samples with 0.05–0.5% ε-PL did not suffer deterioration, suggesting that ε-PL could effectively inhibit the growth of *Bacillus subtilis*. Previous researches have reported the antimicrobial activity of ε-PL in various foods. The addition of 1.5% ε-PL extended lag time (11.61 h) of *S. typhimurium* compared to the control (5.56 h) in pork at 24 °C [45]. Chang et al. [20] found that 0.1–1% ε-PL reduced the *S. typhimurium* population by 0.68–4.93 log CFU/g in roast beef after 7 days at 4 °C. However, some researchers found that ε-PL could exhibit excellent effect at particularly low concentrations. Geornaras and Sofos [46] found that 0.01% ε-PL showed pronounced effect against *S. typhimurium* in TSAYE on day 6 at 4 °C. Furthermore, Geornaras, Yoon, Belk, Smith and Sofos [47] also demonstrated that 0.04% ε-PL reduced the *S. typhimurium* populations to low levels even below the detection limit in fat-free/whole fat milk, beef, bologna, ice and vegetables at 12 °C on day 6 and the antimicrobial activity was influenced by food composition. 

As reported by previous studies, ε-PL has been widely used to control pathogens in high water foods but rarely in LMFs. In this study, ε-PL did not exhibit any antimicrobial activity in undiluted tahini (Figure 4b), possibly due to the limited movement of antimicrobial compounds to the target site in the bacterial cell and the protective effect of high fat content [20].

As shown in Figure 5C and Table 4, 0.05–0.5% CA concentration dependently slowed down the growth rate of *S. montevideo* CICC21588 in diluted tahini at 25 °C within 24 h due to the decrease of pH value (pH 5.53-4.95). The population reached to similar levels of 8.85–9.06 log CFU/g at 52 h in the absence or presence of CA. However, after 7 d, samples with CA suffered deterioration and influenced the *Salmonella* enumeration, indicating that 0.05–0.5% CA had no antibacterial effect on *Bacillus subtilis*. While during the period of 7 days at 4 °C, 0.3% and 0.5% CA had no antimicrobial activity against *S. montevideo* in diluted tahini and kept stable populations of about 6 log CFU/g (Figure 5c, Table 4). Osaili et al. [48] also found that 0.4–0.8% CA had no significant effect on the *S. typhimurium* in eggplant dip samples during storage of 15 days at 4, 10 and 21 °C. In tabbouleh salad, 1% CA exhibited no inhibition against *S. typhimurium* at 21 °C but was effective at 4 °C or 10 °C after 7 days [49]. 

In addition, CA exhibited immediate antimicrobial effect against *Salmonella* in water or food surface possibly due to the lack of protective composition [50,51]. For example, 0.5% CA caused 1.26 log CFU/mL reduction of *Salmonella* in washing water of lettuce residues within 15 min of contact [52].

### 3.5. Synergistic Effects of Thermal Inactivation and Antimicrobials in Undiluted or Diluted Tahini

**Figure 6:**
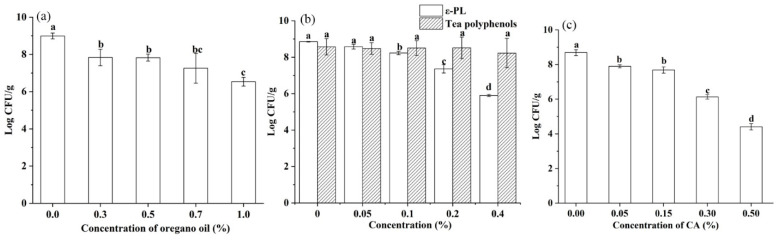
Synergistic antibacterial effects of heating (56 °C for 8 min) and antimicrobials ((**a**): oregano oil, (**b**): ε-PL and tea polyphenol, (**c**): CA) against *S. montevideo* CICC 21588 in diluted tahini at different concentrations. The same low case letter among different concentration treated samples indicates values are not significantly different (*p* > 0.05).

As shown in Figure 6, oregano oil, ε-PL or CA concentration dependently raised the thermal (56 °C for 8 min) inactivation of *S. montevideo* CICC 21588 in diluted tahini. However, the combined treatment of 0.05–0.4% tea polyphenols and heating at 56 °C for 8 min exhibited no synergistic effect on the *S. montevideo* CICC 21588 inactivation (*p* > 0.05). 

**Figure 7:**
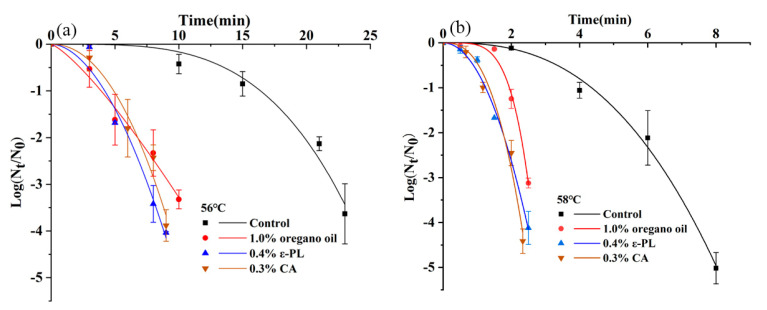
Inactivation curves of *S. montevideo* CICC 21588 in diluted tahini using heating ((**a**): 56 °C or (**b**): 58 °C) combined with oregano oil, CA and ε-PL.

Figure 7 shows the inactivation curves of *S. montevideo* CICC21588 of heating combined with 1% oregano oil or 0.4% ε-PL or 0.3% CA. Table 5 further shows the *δ-* and *p*-values of the Weibull model for thermal inactivation in combination with antimicrobials. The *δ*-values at 56 °C combined with 1% oregano oil, 0.4% ε-PL or 0.3% CA were 3.98 min, 4.20 min and 4.92 min, respectively, while the *δ*-value was 16.46 min by heating alone at 56 °C. Furthermore, the *δ*-values at 58 °C combined with these antimicrobials were also significantly smaller than that of heating alone at 58 °C (*p* < 0.05), indicating that oregano oil, ε-PL and CA all significantly reduced the thermal resistance of *S. montevideo* CICC21588 in diluted tahini.

From Table 6, no synergistic effect was observed at 75 °C with 2% oregano oil. The addition of water-soluble antimicrobials raised the a_w_ of tahini samples, which influenced the thermal resistance of *Salmonella* [31]. The a_w_ increased from 0.256 of original sample to 0.283 or 0.335 of samples with 0.3% CA or 0.4% ε-PL. Therefore, the a_w_ of control group samples was adjusted to the same with antimicrobials treated samples. Although the *δ*-values at 75 °C with 0.3% CA or 0.4% ε-PL significantly decreased as compared to the respective control (*p* < 0.05), significant differences between the population reductions were only observed at 75 °C for 20 min (*p* < 0.05) and no significant difference at 75 °C for 40–100 min (*p* > 0.05), indicating that the addition of CA and ε-PL in undiluted tahini slightly reduced the thermal resistance of *S. montevideo* CICC21588 but the synergistic effects were far lower than those in diluted tahini samples. 

The different synergistic effects obtained between undiluted and diluted tahini may be due to the lower sublethal injured cells caused by thermal treatments in undiluted tahini compared to that in diluted tahini (Figure 3). Antimicrobials could inactivate the sublethal injured cells caused by heating more easily than intact cells through enhanced access to target cell structures [28]. In addition, the synergistic effect may also be the result of the cumulative stresses occurring over a short period, causing multiple damages of bacteria cells so that losing the ability of the bacteria to recover [23]. For example, EOs have been reported to inactivate pathogens through alternating the fatty acid profile of cell membrane, disrupting the cytoplasmic membrane, and reducing the proton-motive force (PMF) [53]. ε-PL could also alter the integrity and permeability of cell membranes of *Escherichia coli* O157:H7 [54]. For organic acids, their undissociated form can easily pass through the bacterial membrane and dissociate, resulting in the reduced pH value of the internal cell, finally leading to the cell death [55]. Furthermore, synergistic effects of heating and antimicrobials were widely observed in food surface, juice, milk, various meat products or other foods with high water content but rarely in oily, low a_w_ paste-like foods, such as tahini, peanut paste or nut paste, due to the limited effect in these foods. Espina et al. [35] reported that the combination treatment of orange essential oil or (+)-limonene and mild heat had a synergistic bactericidal effect on *E. coli* O157:H7 in orange juice. Sun-Ah et al. [56] found that superheated steam combined with lactic acid could be used to inactivate food pathogens on cantaloupe surface. Oregano essential oil and CA also raised thermal sensitivity of *Listeria monocytogenes* in sous-vide salmon [57].

## 4. Conclusions

The present study compared effects of hydrophobic antimicrobials, oregano oil and hydrophilic antimicrobials, ε-PL and CA on *Salmonella* inactivation in undiluted and diluted tahini during thermal treatment and storage. Oregano oil and CA had antimicrobial effects against *S. montevideo* CICC21588 in undiluted tahini during the storage period of 7 days at 4 °C or 25 °C. However, only high concentration of oregano oil (3%) exhibited a satisfactory effect but might cause undesirable flavor. In diluted tahini, oregano oil, ε-PL and CA all could inhibit the growth of *S. montevideo* CICC21588 at 25 °C to varying degrees and ε-PL had the best inhibitory effect. These antimicrobials all enhanced *Salmonella* inactivation effects in diluted tahini during thermal treatment. Further research may focus on developing combination of natural antimicrobials to further enhance synergistic effects and reduce the unfriendly flavor in treated products. In addition, the safety and quality including sensory evaluation of tahini samples with these antimicrobials should be considered in further studies. This study may provide some useful information for thermal inactivation of *Salmonella* in tahini or tahini-based products and selecting effective appropriate antimicrobials for practical pasteurization.

## Figures and Tables

**Table 1:** Fitted *D*-values of the first-order kinetic model and *δ*- and *p*-values of the Weibull model for thermal inactivation of *S*. *Montevideo* CICC21588 inoculated into undiluted or diluted tahini at different temperatures with heating rate of 5 °C/min using TDT HBS.

Samples	Moisture Content (%)	a_w_	Temperature (°C)	First-Order Model			Weibull Model			
				*D* (min)	R^2^	RMSE	*δ*	*p*	R^2^	RMSE
Undiluted tahini	0.629 ± 0.001	0.256 ± 0.002	75	38.43 ± 4.62	0.885	0.435	10.65 ± 1.18	0.47 ± 0.07	0.996	0.078
			85	31.43 ± 1.41	0.856	0.581	5.21 ± 1.71	0.42 ± 0.04	0.994	0.114
			95	19.66 ± 2.74	0.851	0.601	3.84 ± 0.67	0.45 ± 0.05	0.985	0.190
Diluted tahini	50.240 ± 1.290	1.000 ± 0.000	56	7.17 ± 0.76	0.775	0.810	16.52 ± 1.42	3.91 ± 1.21	0.973	0.279
			58	1.67 ± 0.17	0.853	0.913	4.38 ± 0.52	2.70 ± 0.48	0.993	0.195
			60	0.46 ± 0.04	0.906	0.602	0.92 ± 0.08	2.09 ± 0.39	0.985	0.237

**Table 2:** Antimicrobial activity of EOs against 4 *Salmonella* strains using the oxford cup assay.

Foodborne Pathogens	Essential Oils (EOs)								
	Oregano	Cinnamon	Thyme	Tea tree	Eucalyptus	Peppermint	Garlic	Turmeric	Control
*S. montevideo* CICC21588	23.42 ± 1.02 ^a^	22.58 ± 0.92 ^a^	16.28± 0.66 ^b^	12.50 ± 0.41 ^c^	8.75 ± 0.35 ^d^	8.00 ± 0.00 ^d^	8.00 ± 0.00 ^d^	8.00 ± 0.00 ^d^	8.00 ± 0.00 ^d^
*S*. *Typhimurium* CMCC50115	24.00 ± 0.41 ^a^	22.00 ± 0.41 ^b^	20.63 ± 0.48 ^c^	14.38 ± 0.48 ^e^	15.25 ± 0.65 ^d^	8.83 ± 0.29 ^g^	9.76 ± 0.35 ^f^	8.00 ± 0.00 ^h^	8.00 ± 0.00 ^h^
*S. Mbandaka* NYS5-8	21.38 ± 0.48 ^a^	19.88 ± 0.75 ^b^	17.38 ± 0.48 ^c^	13.00 ± 0.00 ^d^	11.00 ± 0.00 ^e^	8.00 ± 0.00 ^g^	9.25 ± 0.35 ^f^	8.00 ± 0.00 ^g^	8.00 ± 0.00 ^g^
*S. enteritidis* R8-1-1	22.38 ± 0.48 ^a^	22.75 ± 0.65 ^a^	16.00 ± 0.41 ^b^	14.25 ± 0.29 ^c^	11.00 ± 0.00 ^d^	8.00 ± 0.00 ^e^	8.00 ± 0.00 ^e^	8.00 ± 0.00 ^e^	8.00 ± 0.00 ^e^

The same lower case letter within a row among different treatments indicates mean values are not significantly different (*p* > 0.05). The antimicrobial activity is expressed by the inhibition zone diameter (mm); the additive amount is 100 μL essential oils/cup.

**Table 3:** Antimicrobial activity of ε-PL, tea polyphenol, citric acid and lactic acid against 4 *Salmonella* strains using the oxford cup assay.

Foodborne Pathogens	Control	ε-PL		Tea Polyphenol		Citric Acid		Lactic Acid	
		10%	5%	10%	5%	10%	5%	10%	5%
*S. montevideo* CICC21588	8.00 ± 0.00 ^f^	23.50 ± 0.71 ^c^	20.50 ± 0.71 ^d^	11.38 ± 0.75 ^e^	8.00 ± 0.00 ^f^	27.61 ± 0.48 ^a^	23.13 ± 0.85 ^c^	26.08 ± 0.38 ^b^	21.00 ± 0.41 ^d^
*S. Typhimurium* CMCC50115	8.00 ± 0.00 ^f^	17.50 ± 0.50 ^d^	12.75 ± 0.65 ^e^	18.50 ± 0.71 ^d^	13.33 ± 0.58 ^e^	25.75 ± 1.19 ^a^	21.38 ± 0.48 ^c^	24.63 ± 0.48 ^b^	20.38 ± 0.48 ^c^
*S. Mbandaka* NYS5-8	8.00 ± 0.00 ^h^	16.25 ± 0.35 ^e^	11.50 ± 0.71 ^g^	14.75 ± 0.35 ^f^	11.75 ± 0.35 ^g^	26.50 ± 0.50 ^a^	22.25 ± 0.50 ^c^	24.00 ± 0.41 ^b^	17.75 ± 0.29 ^d^
*S. enteritidis* R8-1-1	8.00 ± 0.00 ^f^	18.13 ± 0.63 ^c^	15.00 ± 0.41 ^d^	13.00 ± 1.47 ^e^	8.00 ± 0.00 ^f^	27.70 ± 0.68 ^a^	24.09 ± 0.33 ^b^	27.20 ± 0.53 ^a^	23.68 ± 0.35 ^b^

The same lower case letter within a row among different antimicrobial treatments indicates mean values are not significantly different (*p* > 0.05). The antimicrobial activity is expressed by the inhibition zone diameter (mm); the additive amount is 100 μL essential oils/cup.

**Table 4:** Antimicrobial activity of oregano oil, ε-PL and CA against *S. montevideo* CICC 21588 in diluted tahini at 25 °C or 4 °C.

Antimicrobials		Day 0	25 °C				4 °C			
			1	2 (52 h)	3	7	1	2 (52 h)	3	7
Control	0%	6.19 ± 0.05 ^a^	9.30 ± 0.17 ^a^	8.64 ± 0.01 ^cd^	8.70 ± 0.14 ^abc^	DE	6.27±0.13 ^a^	6.15 ± 0.08 ^a^	6.14 ± 0.08 ^a^	6.22 ± 0.19 ^a^
Oregano oil	1%	6.19 ± 0.05 ^a^	9.26 ± 0.06 ^a^	8.88 ± 0.08 ^ab^	8.54 ± 0.04 ^bc^	8.48 ± 0.19 ^c^	6.26 ± 0.09 ^a^	6.07 ± 0.11 ^a^	6.06 ± 0.05 ^ab^	5.83 ± 0.05 ^ab^
	2%	6.19 ± 0.05 ^a^	8.70 ± 0.02 ^b^	8.99 ± 0.17 ^a^	8.93 ± 0.04 ^ab^	8.44 ± 0.06 ^c^	5.52 ± 0.14 ^b^	5.84 ± 0.22 ^b^	5.99 ± 0.02 ^ab^	5.50 ± 0.40 ^b^
	3%	6.19 ± 0.05 ^a^	8.33 ± 0.10 ^c^	8.49 ± 0.07 ^d^	8.67 ± 0.36 ^abc^	8.74 ± 0.04 ^b^	4.70 ± 0.10 ^c^	4.21 ± 0.10 ^c^	4.18 ± 0.32 ^c^	2.63 ± 0.46 ^e^
ε-PL	0.2%	6.13 ± 0.08 ^a^	8.27 ± 0.06 ^c^	8.72 ± 0.12 ^bc^	8.95 ± 0.13 ^a^	9.36 ± 0.004 ^a^	6.13 ± 0.07 ^a^	6.08 ± 0.10 ^a^	6.04 ± 0.03 ^ab^	6.02 ± 0.11 ^a^
	0.4%	6.13 ± 0.08 ^a^	6.21 ± 0.02 ^d^	6.00 ± 0.14 ^e^	7.48 ± 0.25 ^d^	9.29 ± 0.16 ^a^	6.15 ± 0.12 ^a^	5.97 ± 0.11 ^ab^	5.99 ± 0.02 ^ab^	4.58 ± 0.17 ^c^
	0.5%	6.13 ± 0.08 ^a^	5.92 ± 0.11 ^e^	5.88 ± 0.11 ^e^	6.16 ± 0.06 ^e^	9.31 ± 0.04 ^a^	6.13 ± 0.10 ^a^	5.95 ± 0.08 ^ab^	5.88 ± 0.06 ^b^	3.68 ± 0.59 ^d^
CA	0.3%	6.14 ± 0.03 ^a^	9.23 ± 0.03 ^a^	9.02 ± 0.06 ^a^	8.44 ± 0.07 ^c^	DE	6.16 ± 0.05 ^a^	5.99 ± 0.07 ^ab^	6.14 ± 0.11 ^a^	6.03 ± 0.11 ^a^
	0.5%	6.14 ± 0.03 ^a^	8.55 ± 0.03 ^b^	8.85 ± 0.05 ^abc^	8.57 ± 0.04 ^abc^	DE	6.15 ± 0.01 ^a^	6.06 ± 0.04 ^a^	6.12 ± 0.10 ^a^	6.04 ± 0.15 ^a^

The same lower case letter with a column among different antimicrobial treatments indicates mean values are not significantly different (*p* > 0.05). DE means samples suffered deterioration.

**Table 5:** *δ*- and *p*-values of the Weibull model for thermal inactivation combined with antimicrobials against *S. montevideo* CICC21588 inoculated into diluted tahini.

	Temperature (°C)	Weibull Model			
		*δ*	*p*	*R* ^2^	RMSE
Diluted tahini	56	16.52 ± 1.42 ^a^	3.91 ± 1.21	0.973	0.279
	58	4.38 ± 0.52 ^b^	2.70 ± 0.48	0.993	0.195
Diluted tahini+1% oregano oil	56	3.98 ± 0.64 ^b^	1.30 ± 0.21	0.983	0.201
	58	1.94 ± 0.06 ^c^	4.57 ± 0.39	0.994	0.123
Diluted tahini+0.4% ε-PL	56	4.20 ± 0.13 ^b^	1.87 ± 0.02	0.976	0.333
	58	1.25 ± 0.03 ^c^	2.04 ± 0.19	0.991	0.194
Diluted tahini+0.3% CA	56	4.92 ± 1.13 ^b^	2.24 ± 0.73	0.971	0.313
	58	1.35 ± 0.11 ^c^	2.66 ± 0.30	0.980	0.299

The same lower case letter with a column among different samples and temperatures indicates mean values are not significantly different (*p* > 0.05).

**Table 6:** Synergistic effects of thermal inactivation in combination with antimicrobials against *S. montevideo* CICC21588 inoculated into undiluted tahini.

Samples	a_w_	75 °C + Holding Time (min)				Weibull Model			
		Population Reduction (Log CFU/g)							
		20	40	70	100	*δ*	*p*	*R* ^2^	RMSE
Undiluted tahini	0.256 ± 0.002	1.26 ± 0.04 ^a^	1.95 ± 0.13 ^a^	2.43 ± 0.27 ^a^	2.82± 0.38 ^ab^	10.65 ± 1.18 ^a^	0.47 ± 0.07	0.996	0.078
Undiluted tahini+2% oregano oil		1.13 ± 0.01 ^a^	1.79 ± 0.02 ^a^	2.36 ± 0.18 ^a^	2.52 ± 0.21 ^a^	12.48 ± 1.46 ^a^	0.53 ± 0.03	0.986	0.028
Undiluted tahini	0.283 ± 0.012	1.58 ± 0.02 ^b^	1.68 ± 0.10 ^a^	2.84 ± 0.12 ^ab^	3.16 ± 0.10 ^bc^	10.29 ± 0.35 ^a^	0.51 ± 0.01	0.965	0.266
Undiluted tahini+0.3% CA		1.82 ± 0.16 ^c^	2.22 ± 0.02 ^ab^	2.86 ± 0.09 ^abc^	3.32 ± 0.06 ^bc^	4.72 ± 1.26 ^b^	0.39 ± 0.03	0.998	0.059
Undiluted tahini	0.335 ± 0.004	1.81 ± 0.01 ^c^	2.66 ± 0.56 ^bc^	3.37 ± 0.21 ^c^	3.54 ± 0.33 ^c^	3.81 ± 0.96 ^b^	0.40 ± 0.01	0.991	0.160
Undiluted tahini+0.4% ε-PL		2.33 ± 0.14 ^d^	2.84 ± 0.19 ^c^	3.23 ± 0.28 ^bc^	3.48 ± 0.07 ^c^	0.61 ± 0.09 ^c^	0.23 ± 0.03	0.999	0.016

The same lower case letter with a column among different samples indicates mean values are not significantly different (*p* > 0.05).

## Data Availability

The datasets generated for this study are available on request to the corresponding author.
